# Effect of electroacupuncture on diabetic neurogenic bladder: A randomized controlled trial protocol

**DOI:** 10.1097/MD.0000000000019843

**Published:** 2020-04-24

**Authors:** Xuke Han, Yang Gao, Shengju Wang, Qiu Chen

**Affiliations:** a Hospital of Chengdu University of Traditional Chinese Medicine; b Chengdu University of Traditional Chinese Medicine, Chengdu, Sichuan Province, China.

**Keywords:** acupuncture, diabetic neurogenic bladder, protocol, randomized controlled trial, urodynamic tests

## Abstract

**Background:**

The most common and bothersome lower urinary tract complication of diabetes mellitus is diabetic neurogenic bladder (DNB). Acupuncture has certain advantages in treating bladder dysfunction including urinary retention and incontinence. Therefore, we think that electroacupuncture (EA) may be beneficial to DNB patients. However, it is not clear whether EA combined with basic western medicine could optimize the therapeutic effect for DNB.

**Method/design:**

This is a sham-controlled, patient-blinded, pioneer randomized controlled trial (RCT). One hundred fifty eligible patients will be randomly divided into 3 groups: A. basic western medicine (BWC), B. EA with BWC, C. sham EA with BWC. EA treatment will be given twice a week for 12 weeks at bilateral BL23, BL32, BL33, and BL35. The BWC group will received Alpha-lipoic acid (ALA) and methylcobalamin (MC) treatment for 12 weeks, 2 treatment sessions per week. The primary outcome is scored by the 72-hour bladder diary (72h-BD). The secondary outcomes will be scored by the American Urological Association symptom index (AUA-SI), Post-void residual urine volume (PVR) and urodynamic tests. All the assessments will be conducted at baseline and the 12th weeks after the intervention starts. The follow-up assessments will be performed with 72h-BD and AUA-SI in the 4th, 12th, and 24th weeks after intervention ends.

**Discussion:**

This trial protocol provides an example of the clinical application acupuncture treatment in the management of DNB. This RCT will provide us information on the effect of treating DNB patients with only acupuncture, western medicine therapy (ALA + MC) as well as the combination of both. The additive effect or synergistic effect of acupuncture and basic western medicine will then be analyzed.

**Trial registration:**

Chinese Clinical Trial Registry, ChiCTR2000030421.

## 1 Introduction

Normal urination requires proper function of both the bladder and the urethra, the process of micturition is controlled by the central nervous system (CNS). Neurogenic bladder includes any lower urinary tract dysfunction caused by the spinal cord injury (SCI) or the CNS lesion or disease.^[1]^ The neurogenic bladder dysfunction (NBD) is a chronic bladder urethra dysfunction, the clinical manifestations of NBD include micturition dysfunction and urine retention. It is reported that the underlying pathological mechanism of NBD is detrusor overactivity and detrusor sphincter dyssynergia.^[2]^ NBD has a high incidence, and about 50,000 patients with NBD are founded every year in China.^[3]^ Many patients with neurologic conditions are complicated by neurogenic bladder dysfunction. It is estimated that the incidence of NBD is 40% to 90% in patients with multiple sclerosis (MS), 70% to 84% in patients with spinal cord injury, 37% to 72% in patients with parkinsonism and 15% in patients with stroke, some patients with diabetic autonomic neuropathy also have NBD.^[4]^ Diabetic neurogenic bladder (DNB) is a manifestation of diabetic neuropathy in the urinary system. Its main features are impaired sensory nerves in the bladder, decreased detrusor contractility, increased bladder capacity, and increased residual urine volume. Related studies found that patients with DNB in clinic often appear urinary system symptoms including urinary incontinence, frequency, and urgency, and have a higher incidence of urinary tract infection and urinary tract obstruction. Meanwhile, patients with DNB are also at risk of serious systemic illnesses including septicemia and acute renal failure if they are not treated properly.^[5]^ According to literature data, the prevalence of DNB in diabetic patients is about 40% to 80%, and even under the condition of good blood glucose control, its incidence is still 25%.^[6]^ Further research shows that the incidence of DNB in patients with diabetes for more than 10 years is as high as 25%, and that of patients with diabetes over 15 years is as high as 50%.^[7]^

Although the increasing incidence of DNB in the Asia-pacific region, but there is no reliable and effective treatment. Therefore, most of the treatment programs of DNB are conservative intervention, nutritional nerve antioxidant to reduce residual urine volume and prevent upper urinary tract injury, while this basic western medicine (BWM) treatment cannot achieve satisfactory effect. The reason for this is that the rehabilitation and reconstruction of bladder function requires long-term intervention, while patients with DNB are usually discharged after acute phase and in early rehabilitation. They usually lack long-lasting interventions, which may lead to a series of complications such as urinary tract infection, urinary injury, and kidney damage. Therefore, longterm, safe and effective treatment has become an urgent need for DNB patients.

Traditional Chinese acupuncture therapy has been successfully used to treat nervous system diseases for thousands of years.^[8]^ In fact, some research on acupuncture for bladder dysfunction has revealed its advantages,^[9–11]^ include relieving symptoms, improving patient quality of life, and delaying the progression of neurogenic bladder dysfunction. Electroacupuncture (EA) has been found to improve chronic urinary retention, decrease urine leakage.^[12,13]^ we believe that EA might be an effective treatment for option for DNB. Besides, it is still unknown whether the combination of acupuncture and BWM therapies could optimize the therapeutic effect. The aim for this randomized controlled trial (RCT) is to clarify the therapeutic effect of acupuncture combined with BWC for DNB.

The purpose of this study is as follows: The first objective of this study is to determine whether EA is effective as compared to a sham EA control on DNB. The second aim for this RCT is to clarify the therapeutic effect of acupuncture combined with BWC for DNB. The findings of this trial will be shared through publication and will provide useful information in forming an optimal acupuncture treatment protocol for DNB.

## 2 Methods/design

### 2.1 Hypotheses

EA treatment could relieve the urinary system related symptoms and improve the life quality of patients with DNB.

### 2.2 Objectives

1. To compare the differences in improvement of urological symptoms, assessed by the American Urological Association symptom index (AUA-SI) and 72-hour bladder diary (72h-BD), between the EA group and Sham EA group.
2. To compare the differences in improvement of bladder function, measured by the Urodynamic study and ultrasonic post-void residual urine volume (PVR), between the EA group and Sham EA group.
3. To determine the effect of the credibility of acupuncture treatment on the efficacy, as assessed by the credibility of treatment rating scale (CTRS).
4. To analyze the additive effect or synergistic effect of EA and BWM treatment for DNB.

### 2.3 Study design

We propose a single-center, single-blind, randomized, sham-controlled clinical trial to evaluate the efficacy and safety of EA for neurogenic bladder dysfunction in patients with diabetes mellitus. The trial will be performed at the Hospital of Chengdu University of Traditional Chinese Medicine after ethical approval has been obtained from the Institutional Review Board. A total of 150 eligible patients who meet inclusion criteria will be recruited and randomly divided into the acupuncture group (n = 50), sham acupuncture group (n = 50) and the basic treatment group (n = 50) in a 1:1:1 allocation ratio. After 1 week of washout period, patients will accept 12 weeks of intervention, all treatment will be given twice a week. And the follow-up assessments will be performed in the 4th, 12th, and 24th weeks after intervention ends. The primary outcome will be assessed using the 72h-BD. The secondary outcomes will be measured using the American Urological Association symptom index (AUA-SI) and the PVR, and the urodynamic study. All the assessments will be conducted at baseline, and at the 12th weeks after the intervention starts. The follow-up assessments will be performed with 72h-BD and AUA-SI in the 4th, 12th, and 24th weeks after intervention ends. All participants will sign the informed consent before proceeding with the trial.

**Figure 1:**
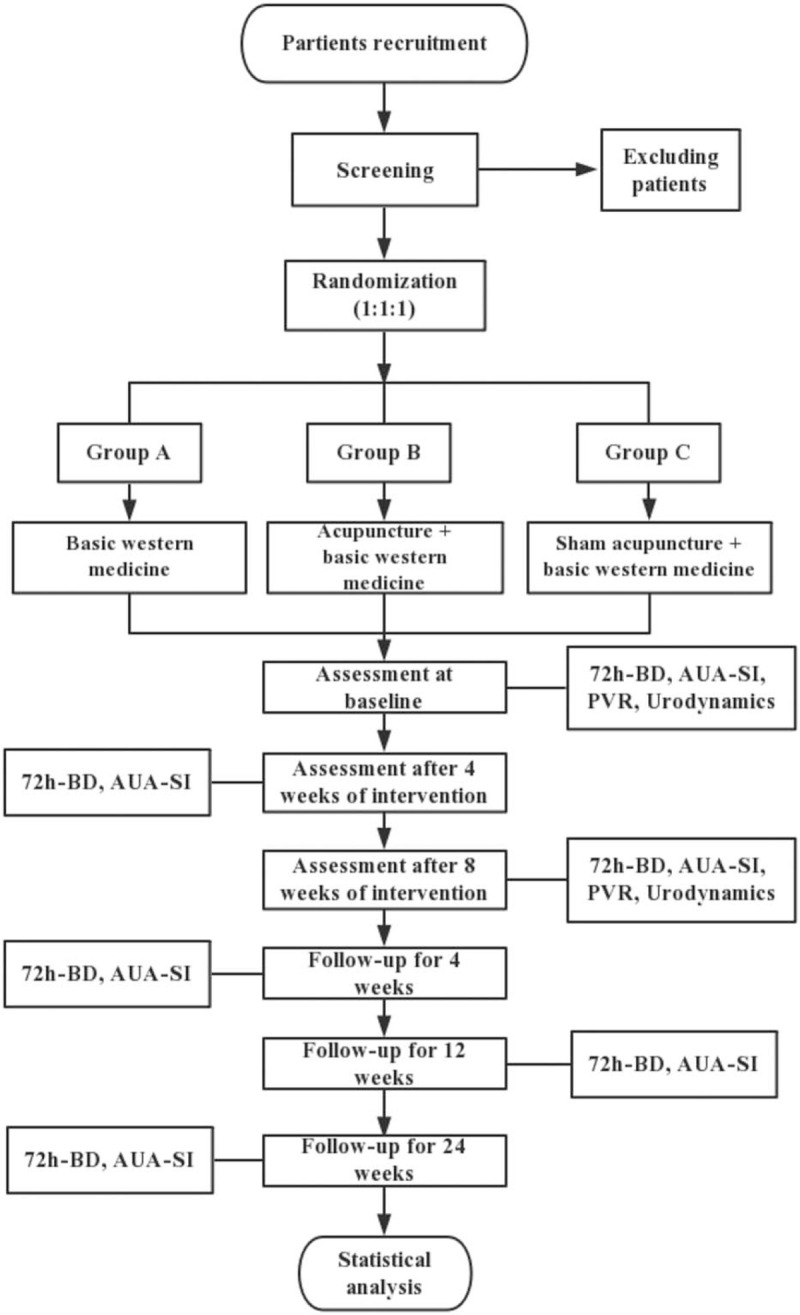
Flowchart of the trial. Figure shows the flowchart of the study process.

The flow chart of the study process is as follows in Figure 1.

And the timing of treatment assessments and data collection are as follows in Table 1.

**Table 1:**
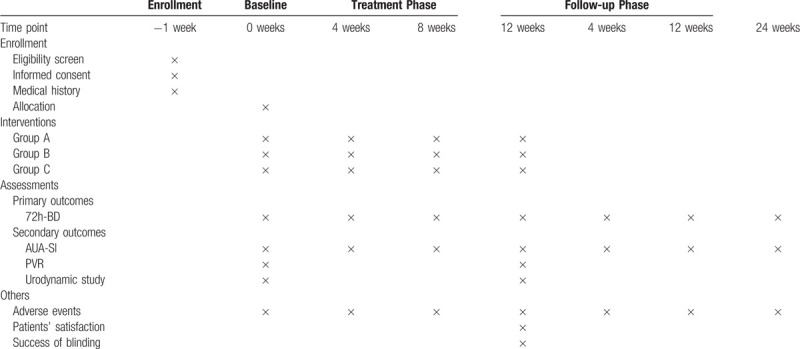
Timing of treatment assessments and data collection.

### 2.4 Recruitment

Participants of the study will be recruited through the outpatient clinic or inpatient, hospital-based Wechat advertising, and posters in Hospital of Chengdu University of Traditional Chinese Medicine in Chengdu, Sichuan Province, China. Any patients who are interested will be screened by telephone or on site and consented for the study. After being informed of the details of this research, participants will be asked to signed informed consent, and deliver it to an independent research assistant. Based on the Western medicine treatment, all participants will receive 12 weeks treatment with acupuncture or sham acupuncture and a series of assessments on bladder dysfunction free of charge.

### 2.5 Inclusion criteria

Participants meeting all of the following conditions will be included:

1. Aged 18 to 75 years; male or female;
2. Participants who meet the diagnostic criteria of diabetes mellitus according to the Standards of medical care for type 2 diabetes in China 2019;^[14]^
3. Participants were detection of bladder PVR by trans-abdominal ultrasound, PVR ≥ 100 mL;^[7]^
4. Participants with complaints of urinary system symptoms at first visit to the doctor;
5. Participants who voluntarily agree with the study and willing to sign the informed consent form for the clinical trial.

### 2.6 Exclusion criteria

Participants meeting any one of the following conditions will be excluded:

1. Participants with severe life-threatening primary disease of the cardiovascular, hepatic, renal and hematopoietic system;
2. Participants with severe mental disorder;
3. Participants had spinal cord injury or other nervous system diseases within the past five years and had undergone pelvic, spinal, and urinary bladder surgery;
4. Participants with urinary calculi, infection or tumor within the past 6 months;
5. Participants who received acupuncture treatment within the past 6 months;
6. Pregnant women.

### 2.7 Sample size

The calculation of the sample size in this trial was based on the study about acupuncture treatment for overactive bladder with urge incontinence.^[15]^ Referring to the study, the designer recruited 85 patients and randomly divided into the acupuncture and placebo acupuncture group. And also used frequency of incontinence episodes from the 72h-BD as the main assessment indicator. To detect a clinically meaningful effect of 30% difference between the acupuncture and placebo acupuncture group, 41 cases will be needed in each group. Under the assumption of a significant level of 0.05, 80% power, and 20% dropout rate, a total of 150 participants should be recruited for this RCT (50 participants in each group with 1:1:1 allocation).

### 2.8 Randomization, allocation, and blinding

The central randomization method will be performed by the Chengdu University of Traditional Chinese Medicine. Block randomization will be used, and the block size will be unknown to the principal investigator (PI) and research personnel. The information of participants will be sent by an independent statistician to the center via the hospital's website. Randomization will be performed automatically under the control of a central computer system. The researcher will be able to get the random numbers and group allocation immediately in the form of an email or short message service. Participants will be randomly divided into 3 groups with the ratio of 1:1:1.

## 3 Intervention

During the study, the patients are permitted to continue their regular medications including lipid-lowering drugs, antihypertensive drugs and drugs not related to the treatment of bladder dysfunction, but no use of the sympathomimetic drugs uracholine or receptor α blockers.

Patients in the 3 groups will receive different treatments of acupuncture and same basic western medicine. Both the acupuncture intervention and western medicine treatment will be given twice a week for 12 weeks, and the follow-up period will be 24 weeks. In each treatment session, every patient will be placed in a separate and quiet space. All the patients will be in prone position and wear an eye-patch. Before all the intervention, the patients’ skin will be disinfected, then the experienced acupuncturist who also be a registered practitioner will begin the treatment.

### 3.1 Group A

Patients in group A only receive basic western medicine (BWM) treatment: 0.9% normal saline (NS) 250 ml + Alpha-lipoic acid (ALA) 600 mg, ivgtt; Methylcobalamin (MC) 0.5 mg, iv; Qd, twice a week for 12 weeks, 24 sessions in total.

### 3.2 Group B

Patients in group B will receive EA with basic western medicine treatment. Pursuant to the traditional Chinese medicine theory, previous relevant studies and the research group acupuncture experts's opinion, the following acupuncture acupoints will be selected (bilateral): BL23 (Shenshu), BL32 (Ciliao), BL33 (Zhongliao), BL35 (Huiyang). Huatuo brand disposable and sterile 0.30 × 75 mm acupuncture needles were used. Following needle insertion, quickly and evenly lift, thrust and rotate the needle to make the patient reports electric shock sensations radiate to the perineum (Deqi sensation). According to the study of EA involving lumbosacral region on stress urinary incontinence,^[12]^ the needles on bilateral BL33 and BL35 will be connected to the acupoint nerve stimulator (SDZ-V; Huatuo Medical Technology Co., Ltd. Suzhou, China), using continuous wave type, with a frequency of 25 HZ, and a current intensity of 1 to 10 mA, resulting in contraction of superficial muscles, which was tolerated by the patients. The EA stimulation lasted for 30 minutes. Participants received 2 treatment sessions per week for 12 weeks, 24 sessions in total. The BWM treatment is the same as group A.

The methods and acupoints for acupuncture intervention are shown in Table 2.

**Table 2:**
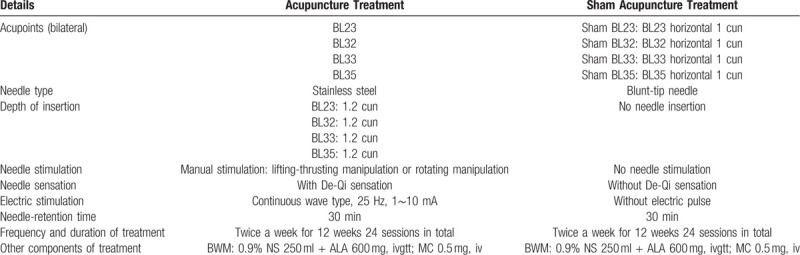
Acupuncture method.

### 3.3 Group C

Patients in group C will receive sham EA with basic western medicine treatment. We use a placebo acupuncture needle that can simulate the acupuncture process and do not penetrate the skin.^[16]^ When the tip of the placebo needle touches the skin, the patient will feel tingling. However, the needle retracts inside the handle and looks shortened as if inserted into the skin.^[17]^ The shame acupoints are as follows: (1)Sham BL 23: BL23 horizontal 1 cun (≈20 mm). (2)Sham BL32: BL32 horizontal 1 cun. (3)Sham BL33: BL33 horizontal 1 cun. (4)Sham BL35: BL35 horizontal 1 cun. Similarly, the acupoint nerve stimulator will be connected to the sham BL33 and sham BL35 (bilateral), without an electricity output for 30 minutes. The BWM treatment is the same as group A.

**Figure 2:**
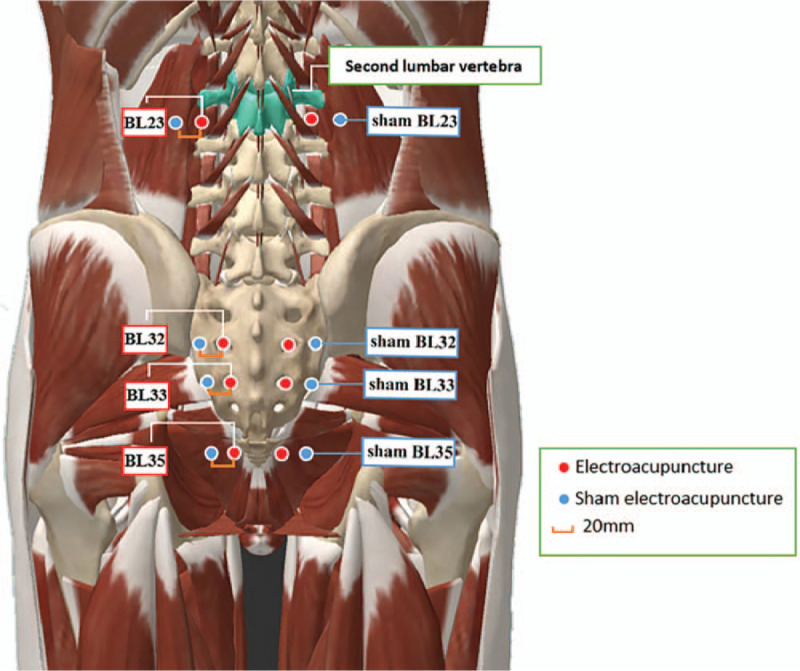
Location of acupoints for the electroacupuncture and sham electroacupuncture groups.

The location of acupoints for the EA and Sham EA Groups are shown in Figure 2.

## 4 Outcomes

### 4.1 Primary outcome

The 72h-BD is a practicable, reliable and effective tool to evaluate the neurogenic bladder related lower urinary tract symptoms.^[18]^ The 72h-BD, which records the frequency of urination, the time of micturition and the urgency of incontinence for 3 days. According to the previous study on this scale to assess bladder-related dysfunction, the frequency of urinary incontinence episodes was selected as the main outcome measurement.^[19]^ The validity of 72h-BD has been validated in patients by the previous clinical studies, and as a non-invasive assessment method, the Chinese version of the scale was verified to be safe and risk-free.^[12]^

### 4.2 Secondary outcomes

#### 4.2.1 American urological association symptom index (AUA-SI)

The American Urological Association Symptom Index (AUA-SI) is a commonly used screening tools assess the overall severity of urinary system disease in patients.^[20,21]^ Clinical studies have confirmed the application value of AUA-SI in DNB-related research and recommend this scale to verify the effectiveness of DNB treatment.^[7,22]^ The AUA-SI contains 7 entries including 4 bladder voiding symptoms (incomplete emptying, weak stream, abdominal straining, and intermittency) and 3 bladder storage symptoms (frequency, urgency, and nocturia). Depending on the frequency of symptoms, each symptom score ranges from 0 (not at all) to 5 (almost always), for a total of 35 points. According to the total score of symptoms, it can be further divided into voiding symptoms score (incomplete emptying + weak stream + intermittency + straining) and storage symptom score (frequency + nocturia + urgency). Importantly, the 8th question of AUA-SI (“If you were to spend the rest of your life with your urinary condition just the way it is now, how would you feel about that? Answer ranging from 0 “delighted” to 6 “terrible”) was used as an overall assessment of a patient's bother regarding their urinary tract condition.^[23]^

#### 4.2.2 Post-void residual urine volume (PVR)

PVR is the amount of urine retained in the bladder after a voluntary void and functions as a non-invasive diagnostic means to assess the stage of neurogenic bladder.^[24]^ The ultrasound (conventional or real-time) is used to visualize the bladder directly. It measures the bladder's volume using the ultrasound machine's internal volume calculations. Related research demonstrate high accuracy of post-void residual using transabdominal ultrasound with automated measurement of bladder volume.^[25]^ The PVR have been used to examine the urinary retention of many conditions including bladder dysfunction and infection/medication induced neurological disease.

#### 4.2.3 Urodynamic study

Urodynamic tests are used to measure the bladder pressure and urine flow rate in order to estimate the neuromuscular function and dysfunction of the urinary tract, and identify the cause of bladder dysfunction. Urodynamics are commonly used in the diagnosis of stress urinary incontinence, overactive bladder syndrome, neurogenic bladder dysfunction.^[26]^ The urodynamic test is important in planning therapy of DNB, a growing number of researchers reported that urodynamics was well tolerated, most researches recommend urodynamics as the choice of DNB study.^[27,28]^ The multi-channel urodynamics is used to determine bladder capacity, contractility, compliance, sensation, and voiding dynamics. The intravesical pressure (Pves), the abdominal pressure to the bladder wall (Pabd) and the detrusor pressure (Pdet), Volume at first desire to void (mL), volume at maximal bladder capacity (mL), maximal urinary flow rate (mL/s), opening pressure (cmH_2_O), opening time (seconds) were measured. And the criterion of urodynamic diagnoses used in this trial follow the criteria recommended by the International Continence Society standardization sub-committee.^[29]^

### 4.3 Adverse events

Acupuncture is generally considered to be a safe treatment.^[30]^ However, any discomfort or symptoms will be assessed. All details of adverse events will be recorded in the case report forms during the trial. The research personnel will interview patients and complete the adverse event report after the treatment. The DSMB and ethics committee will assess any correlation between the adverse event and the intervention, and make a decision on whether the study should proceed. All treatment related adverse are recorded and compared among groups for the purpose of analyzing the safety of EA treatment.

### 4.4 Credibility of treatment rating scale (CTRS)

The credibility of treatment rating scale (CTRS) is a quick and easy to manage scale for measuring treatment expectancy and assessing the rationale credibility of the acupuncture intervention.^[31,32]^ It consists of 4 parts, including the patient's “perceived logic of the treatment,” “confidence in recommending the treatment to their friends who have similar complaints,” and “confidence in the treatment to relieve their complaint,” to derives the 2 predicted factors (cognitively based credibility and relatively more affectively based expectancy). The lower the total score, the greater the confidence in the treatment.

### 4.5 Assessment of blinding

After the treatment session in weeks 12, the patients will be asked to fill in a questionnaire (Table 3). The percentage of patients from each group who believe that they received a true acupuncture treatment will be the principal standard for measuring the subject blinding success rate. We will analyze the difference in the patients blinding success rates among groups.

**Table 3:**
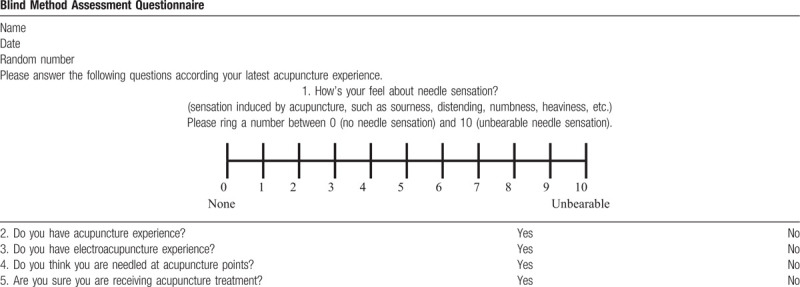
The blind method assessment questionnaire.

The blind method assessment questionnaire is shown in Table 3.

### 4.6 Statistical analysis

The intention-to-treat (ITT) analysis will be used in this study. Data analyses will be performed with the use of the statistical software of SPSS version 23.0. The generalized linear mixed model will be used to compare primary and secondary outcomes between groups and test correlation between baseline factors, and the primary and secondary outcomes. All statistical tests will be two-sided, and *P* < .05 is considered statistically significant.

### 4.7 Monitoring

According to recommendation of the National Institutes of Health (NIH), this trial established an independent Data and Safety Monitoring Board (DSMB). The committee consists of 3 members, including an acupuncturist, an endocrinologist, and a statistician. The committee will supervises whether the study follows the protocol design and standard guidelines, will monitor the progress of the trial, and will observe whether adverse events and etiologies are adequately recorded. This DSMB also identify problems in the project, if any; the committee will make the decisions to change the details of this protocol and announce the research personnel by written notice after approval by the ethics committee. Moreover, a qualified clinical trial expert will be invited to monitor this trial and the PI will take full responsibility and make the final decision.

### 4.8 Ethics approval and consent to participate

This trial was approved by the Ethics Committee of Chengdu University of Traditional Chinese Medicine. Written informed consent will be obtained from all participants prior to enrolment in the study. This RCT will be undertaken according to the ICH-GCP Guidelines. The latest version of this protocol is 1.1, dated 15 December 2019.

### 4.9 Availability of data and materials

All data will be available from the 6th month to the 3rd year after completed and published. For reasonable data requests please contact the corresponding author.

## 5 Discussion

Diabetes mellitus has reached epidemic proportions worldwide, and DNB is a frequent complication related to the duration of diabetes, with a reported incidence ranging from 25% to 87%.^[33]^ These DNB patients may be asymptomatic or have a variety of urinary symptoms, such as overactive bladder and urgency incontinence, resulting in decreased bladder sensation and overflow urinary incontinence.^[34]^ The symptoms of DNB were described as diminished bladder sensation, poor contractility in previous. DNB is currently considered to be a progressive disease including a broad spectrum of lower urinary tract symptoms such as urinary urgency, frequency, incontinence, and nocturia. Although DNB seriously affects the quality of life and threatens the psychological and physical health of individuals, it has received less attention. Usually, DNB patients have reached the advanced stages when they consult a doctor.

To evaluate the effect of urinary incontinence, the bladder diary (safe and convenient, various language version) are recommended by the ICS. The severity of DNB is mainly relied on patient-reported outcomes, and 72 hours voiding diary is widely used as an outcome measure in bladder dysfunction studies. AUA-SI has been validated for patient lower urinary tract symptoms and has now applied to bladder dysfunctions (urgency, frequency, incontinence and nocturia).^[22]^ The diabetic neuropathy can produce voiding dysfunction. And the most reliable examination in patients with a voiding dysfunction is measuring the PVR.^[35]^ Relevant animal and clinical studies show that urodynamic test is an appropriate method to study urinary function.^[36]^ The prevalence of bladder dysfunction diagnosed using urodynamics tests can range from 25% to 90%.^[22]^ Several studies suggest that urodynamic tests could accurately diagnose the underlying pathology in bladder dysfunction.^[37]^

As a feasible complementary and alternative therapy, acupuncture has been widely used in clinical practice in many countries for the treatment of bladder dysfunction and improve neurological functions.^[19]^ In addition, acupuncture is gradually gaining acceptance in urology as an effective treatment for lower urinary tract symptoms.^[38]^ Alpha-lipoic acid (ALA) and methylcobalamin (MC) could improve the signs of diabetic neuropathy by enhancing the metabolism and antioxidant capacity of nerve and vascular endothelial cells.^[39]^ Therefore, MC and ALA are usually selected as clinical basic treatment medicine for DNB. Our study protocol is based on the previous clinical experience and relevant clinic reports. However, this protocol still faces some challenges. The first is the sensitivity of urodynamic test. The urodynamic examination is an invasive test that is influenced by patient emotion environmental factors. The second is the blinding method. The nature of the clinical acupuncture therapy made double-blinding impossible. However, this study still has several strengths. The first innovation of this protocol is use of the CTRS to evaluate patients’ credibility of acupuncture treatment and its impact on acupuncture treatment. Moreover, this is the first study to analyze the curative effect of acupuncture on DNB buy using the objective tools (trans-abdominal ultrasound and multi-channel urodynamics) and subjective method (AUA-SI scale and 72h-BD).

This trial should provide us with information on the effect of treating DNB patients with only EA, western medicine or as a combination of both. The additive effect or synergistic effect of acupuncture and western medicine will then be analyzed. We expect that this trial will advance knowledge on the acupuncture in the treatment of DNB. If the further results confirm that acupuncture therapy is an effective and safe option for DNB, it can be used as a priority treatment in clinical practice.

## 6 Trial status

This trial will start recruiting participants in June 2020, and will be completed by 31 December 2022.

## Author contributions

**Conceptualization**: Qiu Chen, Xuke Han

**Investigation**: Yang Gao

**Supervision**: Shengju Wang

**Writing – original draft**: Xuke Han

**Writing – review & editing**: Qiu Chen, Xuke Han
